# The Infant Gut Commensal *Bacteroides dorei* Presents a Generalized Transcriptional Response to Various Human Milk Oligosaccharides

**DOI:** 10.3389/fcimb.2022.854122

**Published:** 2022-03-18

**Authors:** Sivan Kijner, Avital Cher, Moran Yassour

**Affiliations:** ^1^ Microbiology & Molecular Genetics Department, Faculty of Medicine, The Hebrew University of Jerusalem, Jerusalem, Israel; ^2^ The Rachel and Selim Benin School of Computer Science and Engineering, The Hebrew University of Jerusalem, Jerusalem, Israel

**Keywords:** infant microbiome, human milk oligosaccharides (HMO), *Bacteroides*, microbial RNA seq, breastmilk

## Abstract

Human milk oligosaccharides (HMOs) are a family of glycans found in breastmilk with over 200 identified structures. Despite being t​​he third-largest solid component in breastmilk, HMOs are indigestible by infants, and they serve as food for the infant gut bacteria. Most research thus far has focused on *Bifidobacterium* species that harbor many glycoside hydrolases (GHs) tailored to break the carbon bonds in HMO molecules. However, there are additional microbes in the infant gut, such as *Bacteroides* species, with increasing evidence that they, too, are able to break-down HMOs. To study the unbiased impact of breastfeeding on the infant gut microbiome, we need to investigate the underlying mechanisms of HMO utilization by all members of the infant gut. Here, we developed an optimized system for isolating *Bacteroides* strains from infant stool samples. We then examined the HMO utilization capacity of multiple *Bacteroides* isolates by performing growth curves on six common HMOs (2’-FL, DFL, 3’-SL, 6’-SL, LNT, LNnT). Isolates often displayed similar growth characteristics on similarly-structured HMOs, like sialylated or fucosylated sugars. We identified variation in HMO utilization across multiple strains of the same species, and chose to focus here on a *Bacteroides dorei* isolate that was able to utilize the test HMOs. We performed RNA sequencing on *B. dorei* cultures, comparing the transcriptional profile in minimal media supplemented with glucose or HMOs. We showed that *B. dorei* employs an extensive metabolic response to HMOs. Surprisingly, there was no clear up-regulation for most GH families previously known to break-down HMOs, possibly because they were almost exclusively described in *Bifidobacterium* species. Instead, *B. dorei* exhibits a generalized response to HMOs, markedly up-regulating several shared GH families across all conditions. Within each GH family, *B. dorei* displays a consistent pattern of up-regulation of some genes with down-regulation of the others. This response pattern to HMOs has yet to be described in other commensals of the infant gut. Our work highlights the importance of expanding the HMO-microbiome studies beyond *Bifidobacterium* species, sheds light on the differences across *Bacteroides* strains in terms of HMO utilization, and paves the way to understanding the mechanisms enabling *Bacteroides* HMO utilization.

## Introduction

The infant gut microbiome is very dynamic (Yassour et al., 2016; Ferretti et al., 2018), and stabilizes to an adult-like state around the age of three (Yatsunenko et al., 2012; Yassour et al., 2016). The initial colonization of the infant gut is a complex process (La Rosa et al., 2014; Lax et al., 2014), mainly influenced by delivery mode (Dominguez-Bello et al., 2010; Azad et al., 2013; Madan et al., 2016; Yassour et al., 2016) and infant feeding (formula vs. breast milk) (Azad et al., 2013; Bäckhed et al., 2015; Madan et al., 2016; Stewart et al., 2018). Human Milk Oligosaccharides (HMOs) are a family of glycans found in breast milk, and despite being the third-largest solid component in human milk (Vandenplas et al., 2018), infants themselves are unable to digest these sugars (Engfer et al., 2000; Gnoth et al., 2000), and HMOs are exclusively digested by bacteria in the infant gut. HMOs take part in many important processes, such as infant immune system maturation (Plaza-Díaz et al., 2018) and brain development (Jacobi et al., 2016; Hegar et al., 2019), but one of their most-studied roles is the promotion of a healthy infant gut microbial community, by supporting the colonization of beneficial gut bacteria that can break down the HMO chemical structure (Walsh et al., 2020). Mounting evidence show that early establishment of a “correct” microbial community in the infant gut is crucial for future health, in microbiota-mediated colonization resistance against intestinal pathogens (Buffie and Pamer, 2013; McLaren and Callahan, 2020), even under antibiotic disturbance (Letten et al., 2021a), and the ability of the microbiome to modulate persistence of antibiotic resistant strains (Letten et al., 2021b). The importance of HMOs in shaping the unique infant microbiome has been a subject of avid research in the past two decades, however most of it focused on *Bifidobacterium* species (Dethlefsen et al., 2007; Sela et al., 2008).

*Bifidobacterium* species abundance is strongly associated with HMO consumption, as demonstrated by multiple studies comparing breastfed to non-breastfed infants (Davis et al., 2016; Smith-Brown et al., 2016; Bai et al., 2018; Lawson et al., 2020; Laursen et al., 2021; Liu et al., 2021). The first HMO-specific utilization cluster (H1) was discovered in 2008 (Sela et al., 2008). It is 43kb long and is conserved in all *B. longum subsp. infantis* genomes sequenced thus far. Since 2008, five additional HMO-utilizing gene clusters (H2, H3, H4, H5, urease) have been identified (LoCascio et al., 2010), albeit they are less conserved. Recently, additional genes capable of HMO utilization were discovered, which do not necessarily belong to the known clusters identified thus far, and revealed variation in differential expression of genes across *Bifidobacterium longum subsp. infantis* bacteria (Zabel et al., 2020). Importantly, none of these well-studied gene clusters were found in any non-*Bifidobacterium* species (Sela and Mills, 2010; Marcobal and Sonnenburg, 2012).

Despite the well-established connection between *Bifidobacterium* and HMOs, recent empirical evidence support the HMO utilization by other, non-*Bifidobacterium* species. First, some breastfeeding infants do not harbor any *Bifidobacterium* species throughout their breastfeeding period (Yassour et al., 2016), and findings exhibit inter-study heterogeneity (Harmsen et al., 2000; Favier et al., 2002; Palmer et al., 2007). Importantly, it was recently reported that the best known HMO-utilizer strain, *Bifidobacterium longum subsp. infantis*, considered to be predominant in the infant gut (Chichlowski et al., 2020), is not evident in 90% of infants in the US (Casaburi et al., 2021) and Finland (Vatanen et al., 2019). Second, there are significant correlations between specific HMO levels and the relative abundance of other genera, such as *Lactobacillus* (Bai et al., 2018), *Bacteroides* and *Parabacteroides* (Wang et al., 2015; Borewicz et al., 2019; Borewicz et al., 2020). Specifically, the immediate next candidates for HMO utilization are *Bacteroides* species, which are known degraders of other complex versatile glycans (Hooper et al., 2002; Bjursell et al., 2006; Martens et al., 2008), like mucin coating the colon epithelium (Marcobal et al., 2011; Pruss et al., 2021). When examining the genomes of common infant gut microbes, we can computationally annotate some genes as potential HMO-utilizers (Xu et al., 2003; Yassour et al., 2016; Vatanen et al., 2019), mostly from the *Bacteroides* genus. Lastly, there is experimental evidence that various *Bacteroides* strains can utilize HMOs as their sole carbon source (Salli et al., 2021), and some of the mechanisms have been further characterized (Marcobal et al., 2011). *Bacteroides thetaiotaomicron* and *Bacteroides fragilis* utilized both host mucus and HMOs, using a similar set of upregulated genes (Marcobal et al., 2011). The variety of findings concerning non-*Bifidobacterium* HMO-degraders highlight the importance of expanding our knowledge of HMO utilization among other gut commensals.

We therefore place *Bacteroides* at the heart of this research. First, we developed an optimized system for isolation of *Bacteroides* strains from infant stool samples, utilizing *Bacteroides*-specific qPCR and *Bacteroides*-selective media. Second, we examined the HMO utilization capacity of our *Bacteroides* isolates by analyzing growth curves on six common HMO molecules: 2-Fucosyllactose (2’-FL), Difucosyllactose (DFL), 3-Sialyllactose (3’-SL), 6-Sialyllactose (6’-SL), Lacto-N-Tetraose (LNT) and Lacto-N-neotetraose (LNnT). Isolates often displayed similar growth characteristics on similarly-structured HMOs, like sialylated or fucosylated sugars. We identified variation in HMO utilization across multiple strains of the same species, and chose to focus here on a *Bacteroides dorei* isolate that was able to utilize the test HMOs. We performed RNA sequencing on *B. dorei* cultures, comparing the transcriptional profile in minimal media supplemented with glucose or HMO. We showed that *B. dorei* employs an extensive metabolic response to HMOs: 17 GH families were upregulated when grown on 2’-FL, 21 on DFL, 19 on 3’-SL, 23 on 6’-SL, 15 on LNT, and 18 on LNnT. In addition, *B. dorei* exhibits a generalized response to HMOs, markedly up-regulating several shared GH families across all conditions, in contrast to *Bifidobacterium* species, previously shown to employ specific GH families under different growth conditions. Within each GH family, *B. dorei* displays a consistent pattern of up-regulation of some genes with down-regulation of the others.

## Materials and Methods

### *Bacteroides* Isolation From Stool Samples

Ten stool samples were collected from seven exclusively breastfed infants less than six month old (S4-5, S5-6, S9-10 were collected twice from the same infants with an average of one week interval). Infants were vaginally-born, and did not receive any antibiotic treatment from birth to sample collection time. All seven mothers have agreed to participate in our study, which was approved by the Hebrew University’s Institutional Review Board (IRB), and signed our consent forms. Samples were collected from diapers using a small plastic spoon and tube, stored at 4°C, then transferred to -80°C within 8 hours. The samples were frozen for a maximal time of two months, and thawed twice: once for DNA extraction, and once for plating and *Bacteroides* isolation.

DNA was extracted using the DNeasy PowerSoil Pro Kit (QIAGEN) and used as a template for quantitative PCR (qPCR) reactions, with both general 16S primers (Ivanov et al., 2009; Yassour et al., 2018) (16S_F 5′ GGTGAATACGTTCCCGG 3′, 16S_R 5′ TACGGCTACCTTGTTACGACTT 3′) and *Bacteroides*-specific primers (Matsuki et al., 2002) (g-Bfra-F 5′ ATAGCCTTTCGAAAGRAAGAT 3′, g-Bfra-R 5′ CCAGTATCAACTGCAATTTTA 3′). qPCR reaction volumes were 1 μL 2.5 ng/μL DNA, 0.25 μL 10μM forward primer, 0.25 μL 10μM reverse primer, 3.5 μL Nuclease-free water, 5 μL SYBR Green PCR Master Mix. The amplification program for the *Bacteroides*-specific qPCR consisted of (1) 94°C for 5 min, (2) 94°C for 20s (3) 50°C for 20 s (4) 72°C for 50 s (5) repeat 2 - 4 for 39 times (6) 94°C for 15 s. The amplification program for the general 16S qPCR consisted of (1) 95°C for 10 min, (2) 95°C for 15 s, (3) 60°C for 30 s, and (4) repeat 2 and 3 39 times. The fluorescent products were detected at the last step of each cycle, and reactions were conducted on a BioRad CFX96 Real-Time System.

The cycle threshold (Ct) value was recorded for each sample, and it is inversely proportional to the amount of *Bacteroides* DNA in the sample. The negative control samples (NC1, NC2), that did not contain *Bacteroides* according to previously obtained in-lab 16S metagenomic sequencing, had a high Ct in *Bacteroides-specific* qPCR experiments. In contrast, DNA extracted from a pure *Bacteroides fragilis* (ATCC 25285) culture, serving as a positive control, had a low Ct value.

Samples that passed the initial qPCR screening were plated on blood agar plates (hylabs, PD005) and incubated at 37°C for 48 hours in an anaerobic chamber (COY). Then, colonies were re-streaked onto Bile Esculin Agar (BEA) plates and incubated in the same conditions. PCR reaction for the 16S region was performed using the universal primers 27F AGAGTTTGATCMTGGCTCAG, 1492R GGTTACCTTGTTACGACTT (Lane, 1991). Reaction volumes were 1 μL DNA, 1.25 μL 10μM forward primer, 1.25 μL 10μM reverse primer, 9 μL Nuclease-free water, 12.5 μL SYBR NEB Q5 High-Fidelity MasterMix. The program consisted of (1) 98°C for 30 s, (2) 98°C for 10s (3) 55°C for 15 s (4) 72°C for 30 s (5) repeat 2 - 4 for 29 times (6) 72°C for 2 min. AMPure XP (Beckman Coulter, A63881) bead cleaning of PCR products was performed according to manufacturer’s instructions. Then, Sanger sequencing of the 16S region was performed with the primer 515F GTGCCAGCMGCCGCGGTAA (Caporaso et al., 2011), at Hylabs (Rehovot, Israel). Bacterial identity identification was achieved using BLAST (Altschul et al., 1990) alignment with default settings.

### 16S Ribosomal Gene Sequencing of Stool Samples

Samples that were found to contain *Bacteroides* according to qPCR screening were chosen for 16S rRNA sequencing. 16S rRNA gene sequencing was performed as previously described (Caporaso et al., 2012), and the 16S gene data set consists of sequences targeting the V4 variable region. Sequencing was performed on the Illumina MiSeq platform according to the manufacturer’s specifications, with addition of 5% PhiX, generating single-end reads of 250 bp in length. Human reads were excluded from further processing with Bowtie2 (Langmead and Salzberg, 2012), in addition to quality-based filtering of reads performed using Fastqmcf (Aronesty, 2013). Taxonomic classification was assigned using BURST (Al-Ghalith and Knights, 2020).

### Growth Curves

For each growth curve experiment, an isolate from infant stool samples was plated on a brain heart infusion (BHI) agar plate (Sigma 70138) supplemented with 50 ml/L fetal bovine serum (FBS; Sigma F2442), 10 ml/L trace vitamins (ATCC^®^ MD-VS™), 10 ml/L trace minerals (ATCC^®^ MD-TMS™), 10 ml/L vitamin K1 and hemin (BBL, 212354), 1 g/L D-(+)-Cellobiose (Alfa Aesar, 528507), 1 g/L D-(+)-Maltose (Caisson, 6363537), 1 g/L D-(+)-Fructose (Sigma Aldrich, 1286504), and 0.5 g/L L-Cysteine (Acros Organics, 52904). The individual HMOs were received as a donation from the DSM Nutritional Products Ltd (previously known as Glycom).

The plates were incubated in an anaerobic chamber (COY) for 48 hours, at 37°C. Two single colonies (biological replicates) were transferred to liquid BHI media (Sigma 53286), supplemented as described above, for overnight incubation. These cultures were then diluted 1:100 in *Bacteroides*-specific minimal-media (MM) with 0.5% (w/v) glucose (Martens et al., 2008). After another overnight incubation, the culture was diluted 1:100 into the same *Bacteroides*-specific minimal media, this time with individual HMOs, glucose or lactose as carbon sources (0.5% w/v). All experiments were conducted with two biological replicates and three technical replicates per carbon source, under anaerobic conditions. Growth (OD600) was monitored every 30 minutes for 60h, at 37°C, using the Epoch2 plate reader (Agilent).

Optical density data over time was analyzed using an in-house custom script, the pheatmap (RRID : SCR_016418) and the growthcurver (Sprouffske and Wagner, 2016) packages under R statistical language [R Core Team (2021)].

### RNA Sequencing of *B. dorei* Cultures

Pure cultures of *B. dorei* growing on various carbon sources (in replicates) were harvested at log phase (OD ~ 0.7) using the Direct-zol™ RNA Miniprep Plus kit (Zymo research, R2071). Agilent 2100 Bioanalyzer (Agilent Technologies) was employed for RNA quality control. Samples were processed according to a previously described protocol (Shishkin et al., 2015) and sequenced in two separate pools: HMOs (2’-FL, DFL, 3’-SL, 6’-SL, LNT, LNnT) and glucose. Single-end 75bp sequencing was performed on a NextSeq device.

The raw sequencing data were further filtered by trim_galore (https://github.com/FelixKrueger/TrimGalore), and classification of the *B. dorei* isolate to the strain level was achieved by alignment of reads using BLAST (Altschul et al., 1990). Principal Component Analysis (PCA) of transcriptional profiles on the various carbon sources was performed on the VST (variance stabilizing transformation)-transformed data (Love et al., 2014). Genome mapping to *B. dorei DSM 17855* genome (GenBank: CP046176.1) was performed by Bowtie2 (Langmead and Salzberg, 2012), and differential expression analysis between HMOs and glucose was carried out using featureCounts (Liao et al., 2014) and DEseq2 (Love et al., 2014). Significantly up-regulated genes were selected based on the padj < 0.05 and log2 fold-change > 1 parameters. Gene annotation of glycoside hydrolases (GHs) was downloaded from the CAZy (Carbohydrate Active enZYmes) (Lombard et al., 2014) database. The statistical enrichment of GH genes from multiple families among the up-regulated gene group, compared to all *B. dorei*’s genes, was calculated with hypergeometric test, using the *phyper* function (*stats* package, R version 4.1.1).

## Results

### An Optimized System for Isolation of *Bacteroides* Strains From Infant Stool

**Figure 1:**
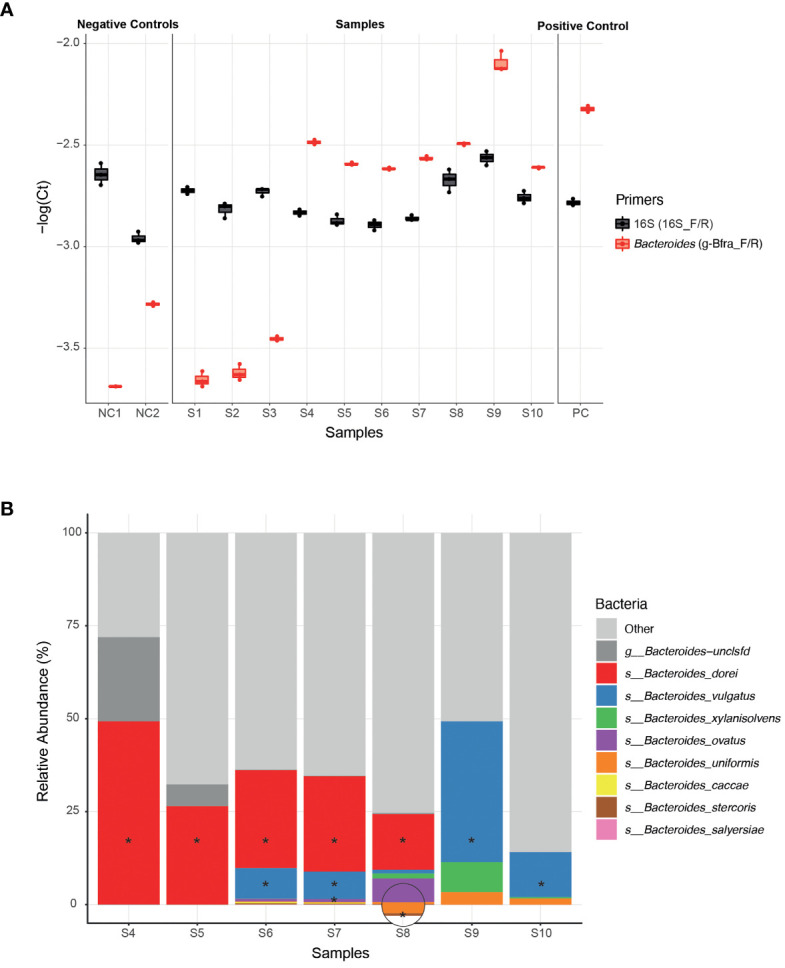
Isolation of *Bacteroides* strains from infant stool samples. **(A)** A comparison of qPCR Ct (Cycle threshold; **Methods**) values of infant stool samples DNA, using *Bacteroides*-specific primers (g-Bfra-F, g-Bfra-R) and universal 16S primers (16_F, 16_R). Each qPCR reaction was performed in triplicates, and each replicate is indicated by a dot. **(B)** Relative abundance of *Bacteroides* species, as identified by 16S rRNA gene sequencing of stool samples found to contain *Bacteroides* according to qPCR screening. *Bacteroides* species that were successfully isolated from stool samples are indicated with asterisks.

To experimentally test the ability of infant *Bacteroides* strains to utilize HMOs as a carbon source, we first need to be able to isolate these infant strains from infant stool samples. In general, the ease of strain isolation differs depending on the species, however, infant stool samples may add an additional layer of complexity due to the low bacterial biomass (compared to adult stool) (Mitchell et al., 2020). Since the isolation process is laborious and time consuming, we aimed to apply it on a selective subset of samples for which we are confident that they harbor *Bacteroides* strains. The earliest possible step to identify the samples containing *Bacteroides* strains is following DNA extraction, for example, by using qPCR. We found that the previously published g-Bfra-F/R (Matsuki et al., 2002) primers enabled a clear distinction between *Bacteroides-*containing and non-*Bacteroides*-containing samples (**Figure 1A**). To control for differences in general bacterial DNA content, we compared the *Bacteroides*-specific Ct value to the universal bacterial 16S Ct value (**Figure 1A**).

Next, we turned to optimize a system for isolation of *Bacteroides* species from samples with probable *Bacteroides* strains. We found that plating stool samples on a rich medium (such as blood agar plates) prior to plating on a more “harsh” selective medium, resulted in enhanced growth of *Bacteroides* and higher isolation success rates, perhaps due to the challenging nature of these *Bacteroides* strains (Medeiros, 1972; Pricop et al., 2020). Thus, we first plated the samples on blood agar plates, then we re-streaked multiple colonies on Bile Esculin Agar (BEA) *Bacteroides*-specific plates (Livingston et al., 1978). BEA plates contain both gentamicin, for which *Bacteroides* are inherently resistant to, and esculin, which *Bacteroides* are able to hydrolyze, producing a black pigment. Then, we picked single black colonies, and performed a second qPCR with *Bacteroides*-specific primers to exclude any non-*Bacteroides* gentamicin-resistant esculin hydrolyzing bacteria that might be present in the stool sample. Finally, Sanger sequencing of the 16S region was performed in order to receive an identification of the bacteria isolated at the genus level.

Indeed, we were able to successfully isolate 11 *Bacteroides* strains from infant samples that passed our qPCR filtering step (S4-S10, **Figure 1A**), whereas no *Bacteroides* were detected after plating and 16S sequencing of the samples that failed the qPCR step (S1-S3, **Figure 1A**).In addition, to test that the isolated strains match the overall *Bacteroides* composition of each sample, we also performed 16S sequencing the samples S4-S10 (**Figure 1B** and **Supplementary Table 1**). All isolated strains belong to species that were identified in the 16S sequencing of each sample. We were able to isolate strains at least from the most abundant species in each sample, and sometimes even from less abundant species, indicating the efficiency of our isolation method (**Figure 1B**).

### Variation in HMO Utilization Across *Bacteroides* Isolates

**Figure 2:**
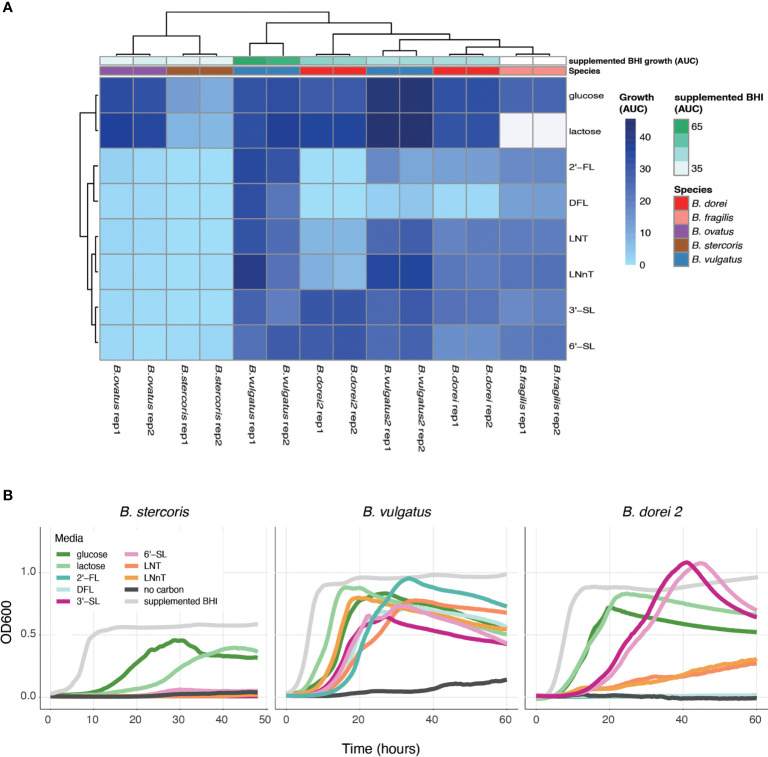
HMO utilization ability varies across *Bacteroides* isolates. Growth curves were measured for all chosen strains and all chosen sources (0.5% weight/volume): glucose, lactose, 2-Fucosyllactose (2’-FL), Difucosyllactose (DFL), 3-Sialyllactose (3’-SL), 6-Sialyllactose (6’-SL), Lacto-N-Tetraose (LNT) and Lacto-N-neotetraose (LNnT), supplemented BHI media (positive control), no carbon media (negative control). **(A)** Area under the curve (AUC) of the growth curve plots, shown in two biological replicates for each strain. Missing data is represented by white cells, in both heat map and annotations. **(B)** Growth curve plots of *B. stercoris*, *B. vulgatus* and *B. dorei 2* on all selected carbon sources.

Once we have established a strain collection composed of all successfully isolated *Bacteroides* strains from infant stool, we next wanted to examine the growth of the unique strains on different complex carbon sources. While there are many types of HMOs, in our study we use synthetic versions of HMOs from all three sub-types, including neutral, fucosylated and sialylated sugars: Lacto-N-Tetraose (LNT), Lacto-N-neotetraose (LNnT), 2′-fucosyllactose (2′-FL), difucosyllactose (DFL), 3-Sialyllactose (3’-SL), and 6-Sialyllactose (6’SL). Growth curve assays on some of the isolated strains were performed with *Bacteroides*-specific minimal media (MM) mixed with the chosen carbon source to a final concentration of 0.5% (weight/volume). Our results indicate that our isolated *Bacteroides* strains differ in their HMO utilization capability and dynamics (**Figures 2A, B** and **Supplementary Figure 1**). Some strains do not grow on any tested HMO as a single carbon source (*B. stercoris*), whereas others exhibit growth on various sugars (*B. doeri 2, B. vulgatus*; **Figures 2A, B** and **Supplementary Figure 1**). Furthermore, we also observed intra-species variation, in both *B. vulgatus* and *B. dorei* strains, where two strains of the same species exhibit distinct utilization patterns. As one might expect, although it has not been reported yet, strains tended to display similar growth characteristics on similarly structured HMOs, for example, the growth pattern of *B. dorei 2* on 3’SL is similar to its growth on 6’SL, and these sugars are both sialylated (**Figure 2B**).

To make sure that the strains cannot grow on any of the minimal media (MM) components on its own, the negative control in our growth experiments was MM without an added carbon source. The positive control was supplemented BHI media, which is a rich nonselective media that supports the growth of many bacteria. Some strains are inherently more difficult to grow in the lab, and thus we used the positive control to evaluate the ease of growth for each strain. In addition, we also measure the strains’ ability to grow on glucose and lactose (the main carbohydrate in breast-milk) (Ballard and Morrow, 2013). Growth experiments can be evaluated by the kinetics of growth (using the OD600 over time), and by emphasizing the quantitative ability of a strain to utilize a carbon source (using the area under the OD600 curve; AUC; **Figure 2A**). The AUC statistic was calculated for all experiments, varied across carbon sources, yet showed consistency across biological replicates (**Figure 2A**).

### Searching for *B. dorei’s* Differentially Expressed Genes Across Multiple HMO Conditions

Differential expression of genes under various conditions can shed light on the enzymes, mechanisms and pathways enabling the utilization of the specific carbon sources. We next chose the isolate of *Bacteroides dorei* that was able to utilize all HMOs tested to some extent, *B. dorei 1* (**Supplementary Figure 1**), to search the genes that enable this utilization. We want to compare the transcribed genes in each condition to identify the upregulated genes that can account for the HMO utilization capability. We grew *B. dorei* on the full set of HMOs described above and harvested the cultures during the mid-log phase to perform transcriptional profiling using RNA sequencing (RNA-Seq).

**Figure 3:**
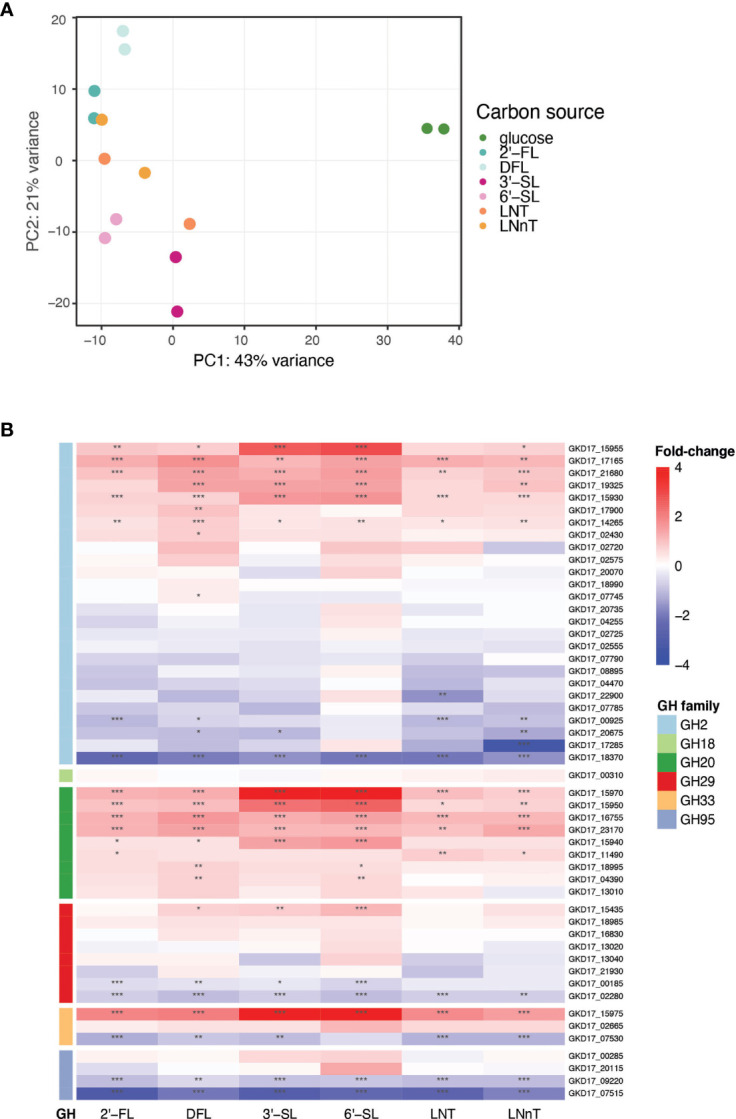
*B. dorei’s* transcriptional response to HMOs is similar across conditions. **(A)** Principal Component Analysis (PCA) of transcriptional profiles of pure cultures of *B. dorei* on various carbon sources, in two biological replicates, using RNA-Sequencing (Methods). **(B)** Heat map of log2 fold-change of genes (rows) belonging to glycoside hydrolase (GH) families previously known to break down HMOs. Gene expression data on HMOs (2’-FL, DFL, 3’-SL, 6’-SL, LNT, LNnT; columns) was compared to gene expression on glucose. p < 0.05 (*); p < 0.01 (**); p < 0.001 (***).

To identify the genes up regulated by *B. dorei* during HMO consumption, whole-genome transcriptional profiling of *B. dorei* on MM supplemented with individual HMOs was compared to growth on MM-glucose (**Supplementary Table 2** and **Supplementary Figure 2**). The two biological replicates of each condition were similar to one another, and more similar within a condition than across conditions (**Supplementary Figure 3**). Fucosylated sugars (2’-FL, DFL) clustered separately from sialylated (3’-SL, 6’-SL), and HMO core structures (LNT, LNnT) clustered in-between (**Figure 3A**). A possible explanation could be that LNT and LNnT lack unique sialyl or fucosyl residues distinguishing them from the other sugars. Overall, the examined conditions are relatively similar, as the tested sugars differ from one another by small modifications only. Thus, we expected the overall gene-expression profiles to be rather similar, with small, yet distinct differences, across all conditions (**Supplementary Figure 3**).

*B. dorei* possesses a repertoire of predicted glycoside hydrolases (GHs), carbohydrate active enzymes (CAZy) capable of breaking the carbon bond found in all human milk oligosaccharides. *B. dorei’s* 213 glycoside hydrolases comprise 59 unique GH families (Lombard et al., 2014). Differential expression analysis of the RNA-Seq data revealed that *B. dorei* exhibits an expansive glycoside hydrolase response during consumption of HMOs *in vitro*. Many unique GH families were upregulated (log2foldChange > 1, padj < 0.05) when growing on HMOs compared to glucose: 17 GH families were upregulated when grown on 2’-FL, 21 on DFL, 19 on 3’-SL, 23 on 6’-SL, 15 on LNT, and 18 on LNnT.

First, we examined the differential expression of GH families previously annotated as capable of breaking down the HMO carbon bond (Marcobal et al., 2011): GH2, GH18, GH20, GH29, GH33, and GH95 (**Figure 3B**). Two additional GH families have been reported in the literature (GH85, GH112) (Ioannou et al., 2021), however they are not encoded in the *B. dorei* genome. Most research regarding HMO utilization by infant gut microbes has focused on *Bifidobacterium* species, hence the knowledge on *Bacteroides* cellular response to HMOs is rather limited. Indeed, some GH families that are well annotated in *Bifidobacterium* species did not exhibit any clear differential expression here, like GH18 and GH95. Surprisingly, some of the GH95 and GH29 *B. dorei* genes, which break the α1-2 and α1-3/4 fucosyl bonds, accordingly, were up-regulated in non-fucosylated HMOs. This lack of specificity is unexpected as we would expect GH95 and GH29 to be upregulated only on fucosylated HMOs. Similarly, GH33 which is responsible for sialic acid release from sialylated HMOs, was up-regulated across all HMO conditions (sialylated and non-sialylated HMOs), although the up-regulation in sialylated oligosaccharides is the most prominent. Lastly, in GH2, the largest GH family in *B. dorei*’s genome (Lombard et al., 2014), some genes were up-regulated while others were down regulated, which might be confusing, and is further discussed below.

### Multiple Glycoside Hydrolase Families Are Upregulated During *B. dorei’s* Growth on HMOs

**Figure 4:**
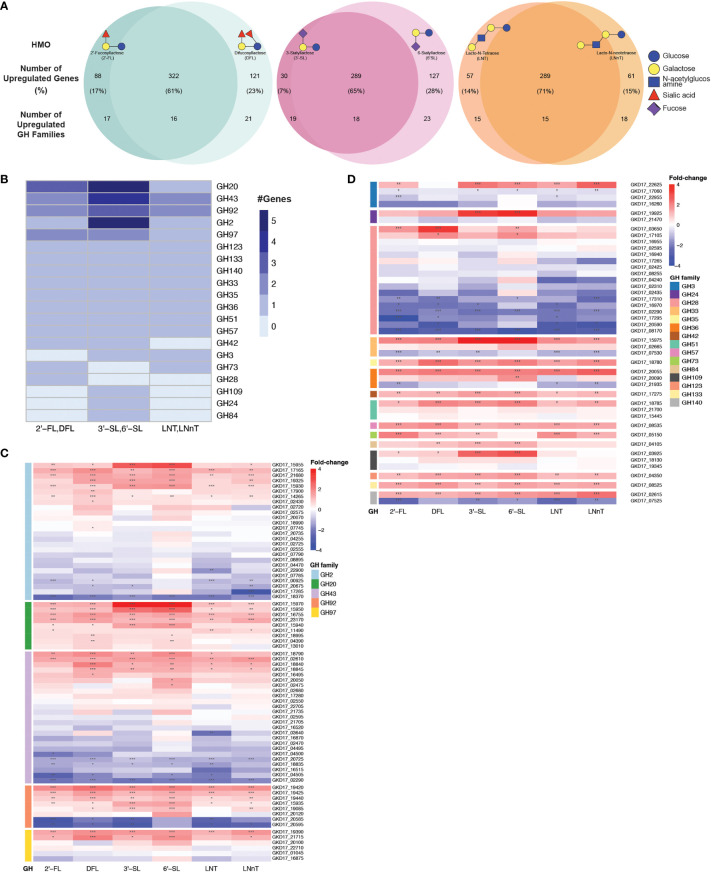
*B. dorei* shares up-regulated genes upon growth on HMOs compared to glucose. **(A)** Venn diagrams of *B. dorei*’s up-regulated genes and glycoside hydrolase (GH) families across HMO types, highlighting the similar transcriptional response to HMOs, regardless of specific conditions. HMOs were divided into pairs based on structural similarity. Percentage values refer to the fraction of up-regulated genes from the total up-regulated genes in both members of an HMO pair. **(B)** A heat map showing the number of up-regulated genes in each up-regulated GH family in at least one HMO pair. **(C, D)** Heat maps of log2 fold-change of genes (rows) belonging to GH families with **(C)** at least two; or **(D)** a single up-regulated gene(s). Gene expression data on HMOs (2’-FL, DFL, 3’-SL, 6’-SL, LNT, LNnT; columns) was compared to gene expression on glucose. p < 0.05 (*); p < 0.01 (**); p < 0.001 (***).

We next performed an unbiased search for all up-regulated GH families in our data, regardless of their annotations. To identify the GH families that could be responsible for *B. dorei*’s HMO utilization, we searched for all GH families that were upregulated in similarly-structured HMO-pairs: 2’-FL and DFL, 3’-SL and 6’-SL, LNT and LNnT (**Figure 4A**). A GH family that is upregulated in two structurally-similar HMOs is more likely to play a role in breaking down these common carbon bonds.

First, we focused on GH families that had a minimum of two upregulated genes for at least one HMO-pair, and found five such GH families: GH2, G20, GH43, GH92, and GH97 (**Figure 4B**). We then expanded our analysis to all genes that belong to these families to compare their regulation across the various HMO structures (**Figure 4C**). We did not find a specific GH family response for a specific HMO-pair, rather, it seems that *B. dorei*’s response to growth on various HMOs is quite generalized, regardless of the specific HMO molecule. Furthermore, even though the response is not specific towards a single HMO, it is specific in the transcriptional changes. Namely, in most cases, only a small subset of the genes in each GH family were up-regulated, and the others were often even down-regulated, suggesting that a specific set of genes, rather than all genes in a certain GH family, responded to the change in carbon source. For example, we found that among *B. dorei*’s 27 GH2 genes, two genes only (GKD17_17165, GKD17_15930) were consistently, significantly upregulated for all conditions. Likewise, for GH43 and GH97, GKD17_02610 and GKD17_19390 were the only consistently upregulated genes, respectively (**Figure 4C**).

Second, we were interested to inspect if the pattern of simultaneous up- and down-regulation of different genes from the same GH family is evident for additional GH families. We looked at GH families for which only a single gene was upregulated in at least one HMO-pair (**Figure 4D**), as a single up-regulated gene can be sufficient for HMO utilization. Similarly to the analysis in the first step (**Figure 4C**), oftentimes, for each GH family, only one or two specific genes were consistently up-regulated, while other members of the family were down-regulated compared to MM-glucose. This observation of specificity in upregulated genes was also evident in smaller GH families, such as GH3 (GKD17_22625), GH33 (GKD17_15975), GH36 (GKD17_20055), GH51 (GKD17_18785). Lastly, GH140 with only two genes presented an interesting pattern, where one was strongly upregulated (GKD17_02615) while the other was strongly down-regulated (GKD17_07525), which may merit further study of this particular GH family. Interestingly, when looking at all GH28 genes encoded in *B. dorei*’s genome, our results suggest that all conditions invoke a strong down-regulation response for many of the GH genes. This consistent broad down-regulation might indicate the up-regulation of other, yet to be annotated, GH genes.

Additionally, enrichment analysis of the shared upregulated GH families revealed that some GH families were enriched in the upregulated gene group compared to the total amount of genes in *B. dorei*’s genome: GH20, GH43 and GH92 were significantly (p<0.05) enriched for DFL, 3’-SL and 6’-SL, GH3 was significantly (p<0.05) enriched for LNT, LNnT, 3’-SL and 6’-SL, GH2 was significantly (p<0.05) enriched for 3’-SL and 6’-SL, and GH36 was significantly (p<0.05) enriched for 6’-SL.

## Discussion

In our quest to investigate HMO utilization by *Bacteroides* infant strains, we have established a system for screening, culturing, measuring growth curves and performing RNA sequencing of *Bacteroides* isolates. We constructed growth curves of *Bacteroides* isolates from infant stool and demonstrated variation in HMO-utilization patterns across *Bacteroides* species and within strains of the same species. Some *Bacteroides* isolates do not grow on HMOs at all, whereas others can utilize all tested HMOs to some extent. This is consistent with previous studies, showing that *Bacteroides* are metabolically diverse and are capable of utilizing many versatile carbon sources (Salyers et al., 1977; Sonnenburg et al., 2005; Bjursell et al., 2006; Marcobal et al., 2011; Wexler and Goodman, 2017; Carrow et al., 2020; García-Bayona and Comstock, 2020; Fultz et al., 2021). Pioneering work in *Bacteroides* demonstrated *B. thetaiotomicron*’s ability to expand and adapt its metabolism, from mainly utilizing glucose and lactose, to also being able to break down more complex plant polysaccharides (Bjursell et al., 2006). Thus, species isolated from infant stool at different time points might alter their metabolic repertoire as the infant ages. A strain’s ability to utilize a wide variety of HMOs may confer a selective advantage over strains that do not utilize these sugars, and could select for its survival in the infant gut.

Our growth curve results demonstrate similar growth patterns on similarly structured HMOs, which may suggest unique carbohydrate utilization pathways being activated for HMOs with similar residues. *Bacteroides* are known to have a wide range of polysaccharide utilization loci (PUL) that encode common classes of proteins involved in polysaccharide utilization, and a large percentage of their genome is dedicated to the sensing, import, and hydrolysis of diverse glycans (Bjursell et al., 2006; Martens et al., 2008). However, RNA-Seq experiments do not show unique genes upregulated under specific conditions. When examining differential expression of glycoside hydrolase (GH) genes of various families, a generalized HMO response was observed, with several GH families upregulated across all six HMOs tested: 2’-FL, DFL, 3’-SL, 6’-SL, LNT, and LNnT. Hence, our findings suggest a unique HMO-utilization strategy among some *Bacteroides* strains, diverging from the extensively studied *Bifidobacterium* HMO utilization mechanisms, where distinct enzymes were recognized for specific carbon bonds in HMO molecules. A possible explanation for the similar response of *B. dorei* to all tested HMOs could be that overall, the conditions tested are rather close, with identical minimal media composition for all samples and small changes in HMO modifications.

*Bifidobacterium* species are considered to be well-adapted to HMO utilization, and they have evolved two distinct strategies for this purpose. One is transporter-dependent (intracellular digestion strategy), and the other is extracellular glycosidase-dependent (extracellular digestion strategy), utilizing the same enzymes, only in a membrane-bound form (Sela et al., 2008; Turroni et al., 2014; Bunesova et al., 2016). Both strategies employ specific enzymes (Ioannou et al., 2021), some of them clustered in specific genomic regions (Sela et al., 2008; LoCascio et al., 2010) whereas others are spread across additional genomic locations (Zabel et al., 2020). In contrast, very little is known about *Bacteroides* HMO utilization, and only a few papers have discussed this issue in terms of phenotypic growth (Salli et al., 2021) or transcriptional profiling (Marcobal et al., 2011). Due to the high-prevalence of *Bacteroides* in the infant gut and a seeming deviation from the traditional *Bifidobacterium*-dominated infant gut microbiome (Vatanen et al., 2019; Casaburi et al., 2021), it is increasingly important to decipher the HMO utilization strategies employed by *Bacteroides* species as well.

*B. dorei*’s differential expression pattern in response to HMOs does not appear to be HMO-specific, however, we show that for the upregulated GH families in our data, a particular set of genes is up-regulated among all members of the GH family encoded in the genome. Moreover, the remaining members of the GH families explored are down-regulated, perhaps meaning that *B. dorei*’s response to HMOs is tailored to exploit some GH genes over others. The novel pattern of HMO utilization presented here can indicate that for a given *Bacteroides* strain, we can discuss specific genes responsible for HMO break-down, in addition to discussing the results on the broader, less-selective, GH family level.

The pioneering work of exploring HMO utilization mechanisms by the type strains of *B. thetaiotaomicron* and *B. fragilis* (Marcobal et al., 2011) found that certain genes are heavily upregulated upon growth on HMOs. These genes belong to several GH families: GH2, GH18, GH20, GH29, GH33, and GH95. There are a few notable differences between this early study and our results. First, the researchers used an HMO mixture, while we used individual HMO molecules. Second, to our knowledge, no study has looked at the transcriptional response of *Bacteroides* strains that were isolated from infant sool to HMOs, which could be different from the type strain response, due to large variation across *Bacteroides* strains of the same species. Lastly, in the 2011 manuscript, the transcriptional response was examined on a pre-selected set of genes (using microarrays designed to capture these specific RNA molecules), whereas we used an unbiased approach of total RNA sequencing. These differences make it difficult to compare the results of both studies, highlighting the need for additional research in additional species, preferably in an unbiased manner.

Notably, the *Bacteroides* genus is characterized by great variations in carbon-utilization abilities, both across species and within strains of the same species (Lange et al., 2016; Husain et al., 2017; Pasolli et al., 2019). Therefore, our findings, exploring one isolate only, do represent a general HMO utilization pattern, and every *Bacteroides* strain or isolate of interest should be individually examined. Second, we compared gene expression profiles of *B. dorei* cultures grown with various HMOs as a single carbon source, in comparison to MM-glucose only. While glucose is the most common reference in microbiology literature, follow up studies should also include galactose and lactose as a reference. Lastly, we should keep in mind that bacteria in the infant gut are simultaneously exposed to multiple types of HMOs, in the context of a complex microbial ecosystem. Experiments such as the ones presented test the utilization capacity of individual strains with a specific HMO molecule. These studies are critical stepping stones towards constructing a comprehensive map of how the infant gut microbes can utilize glycans found in breastmilk, thus shedding light on how breastfeeding, and breastmilk composition, impact the nursing infant gut microbiome.

## Data Availability Statement

The data presented in the study are deposited in the National Center for Biotechnology Information (NCBI) repository, accession number PRJNA804725.

## Ethics Statement

The studies involving human participants were reviewed and approved by Hebrew University’s Institutional Review Board (IRB) (approval number 20042021). Written informed consent to participate in this study was provided by the participants’ legal guardian/next of kin.

## Author Contributions

SK - Established the experimental system, performed experiments and analysis, and wrote the manuscript. AC- Assisted in establishing the experimental system. MY- Guided the work and wrote the manuscript. All authors contributed to the article and approved the submitted version.

## Funding

This work was funded in part by the Azrieli Foundation grant for faculty fellows. SK, AC, and MY were supported by the Azrieli Foundation and MY was also supported by the Israel Science Foundation grant 2660/18.

## Conflict of Interest

The authors declare that the research was conducted in the absence of any commercial or financial relationships that could be construed as a potential conflict of interest.

## Publisher’s Note

All claims expressed in this article are solely those of the authors and do not necessarily represent those of their affiliated organizations, or those of the publisher, the editors and the reviewers. Any product that may be evaluated in this article, or claim that may be made by its manufacturer, is not guaranteed or endorsed by the publisher.

## Supplementary Material

Supplementary Figure 1
**(A)** Growth curves of *B. dorei*, *B. vulgatus 2*, *B. ovatus*, and *B. fragilis* on various HMOs (2’-FL, DFL, 3’-SL, 6’-SL, LNT and LNnT), glucose, lactose, supplemented BHI media (positive control), and no carbon media (negative control). **(B)** Principal Component Analysis (PCA) of *Bacteroides* isolates based on the ability to grow on all media types, as measured by the area under the curve (AUC) of each growth curve.

Supplementary Figure 2 MA plots of HMO transcriptional profiles compared to glucose, shown as M (log ratio) on the y-axis and A (mean average) on the x-axis. Here, the log ratio is calculated as the log2 of the change in expression values of the HMOs vs. glucose, and the mean average is of the glucose read counts. Genes from GH families are labeled with larger dots and display the GH family they belong to. The plot highlights up-regulated (red) and down-regulated (blue) genes, using a threshold of 1 for the absolute log2 fold change, and a threshold of 4 for the log2 glucose mean read count.

Supplementary Figure 3
*B. dorei*’s transcriptional profile, as characterized by RNA-Seq experiments, is similar across conditions. The scatterplots display the normalized read count value for all annotated genes in *B. dorei*’s genome (dots), compared between replicates of the same carbon source (diagonal plots) and between different carbon sources (bottom triangle). Pearson coefficient was calculated for each comparison.

Supplementary Table 1 Bacterial relative abundances (%) as detected by 16S ribosomal RNA gene sequencing, at the species level.

Supplementary Table 2
*B. dorei*’s transcriptional response upon growth in minimal media (MM) supplemented with various HMOs, compared to MM-glucose. Each sheet represents a different HMO compared to glucose, and the last sheet contains explanations of all columns in previous sheets.
