# Supplementary figures and images for: The Infant Gut Commensal Bacteroides dorei Presents a Generalized Transcriptional Response to Various Human Milk Oligosaccharides
Source: Front Cell Infect Microbiol. 2022 Mar 18;12:854122. doi: 10.3389/fcimb.2022.854122 (PMC8971754; doi:10.3389/fcimb.2022.854122)

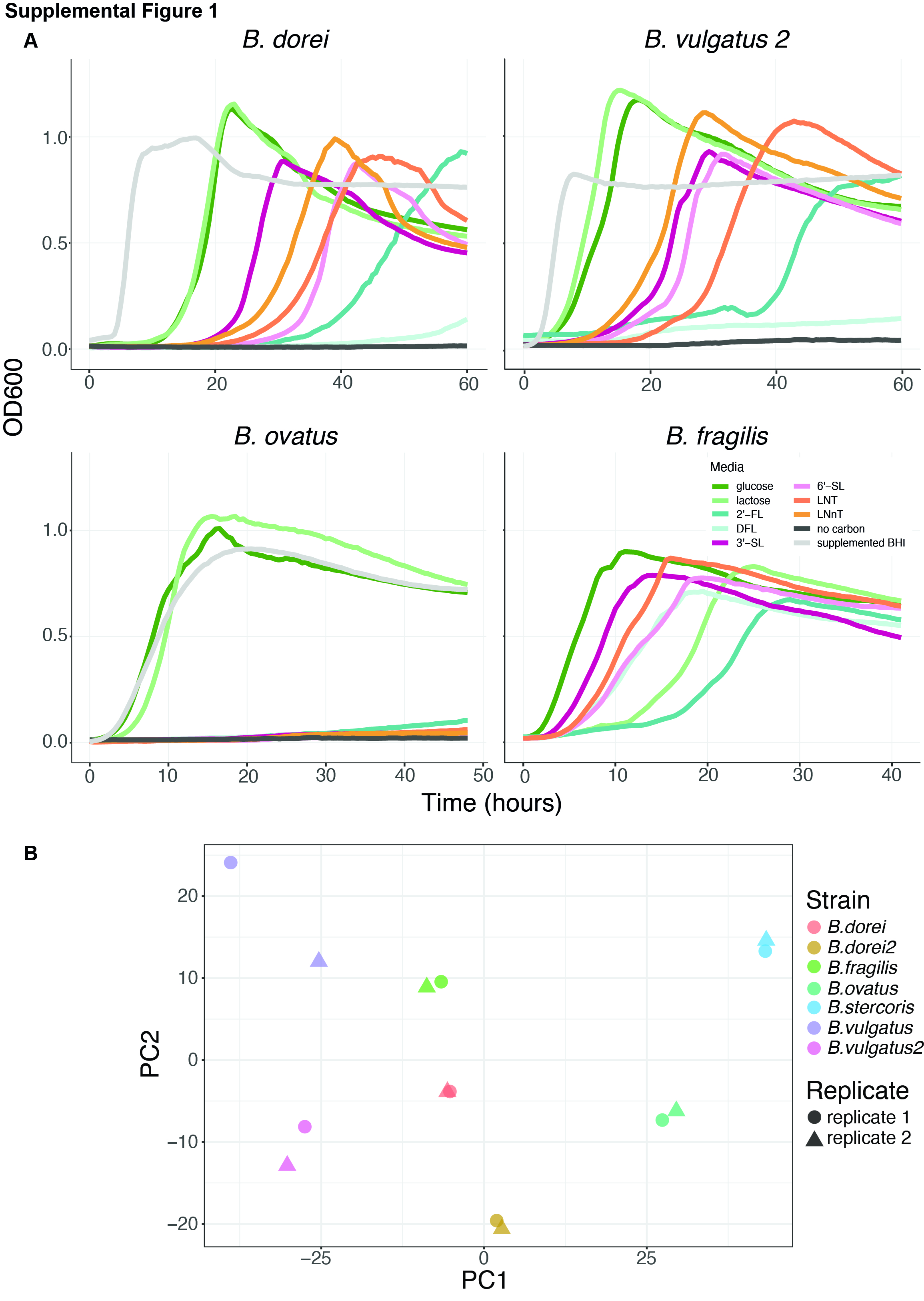

Supplement: Supplementary Figure 1 — (A) Growth curves of B. dorei, B. vulgatus 2, B. ovatus, and B. fragilis on various HMOs (2’-FL, DFL, 3’-SL, 6’-SL, LNT and LNnT), glucose, lactose, supplemented BHI media (positive control), and no carbon media (negative control). (B) Principal Component Analysis (PCA) of Bacteroides isolates based on the ability to grow on all media types, as measured by the area under the curve (AUC) of each growth curve. [file Image_1.jpeg]

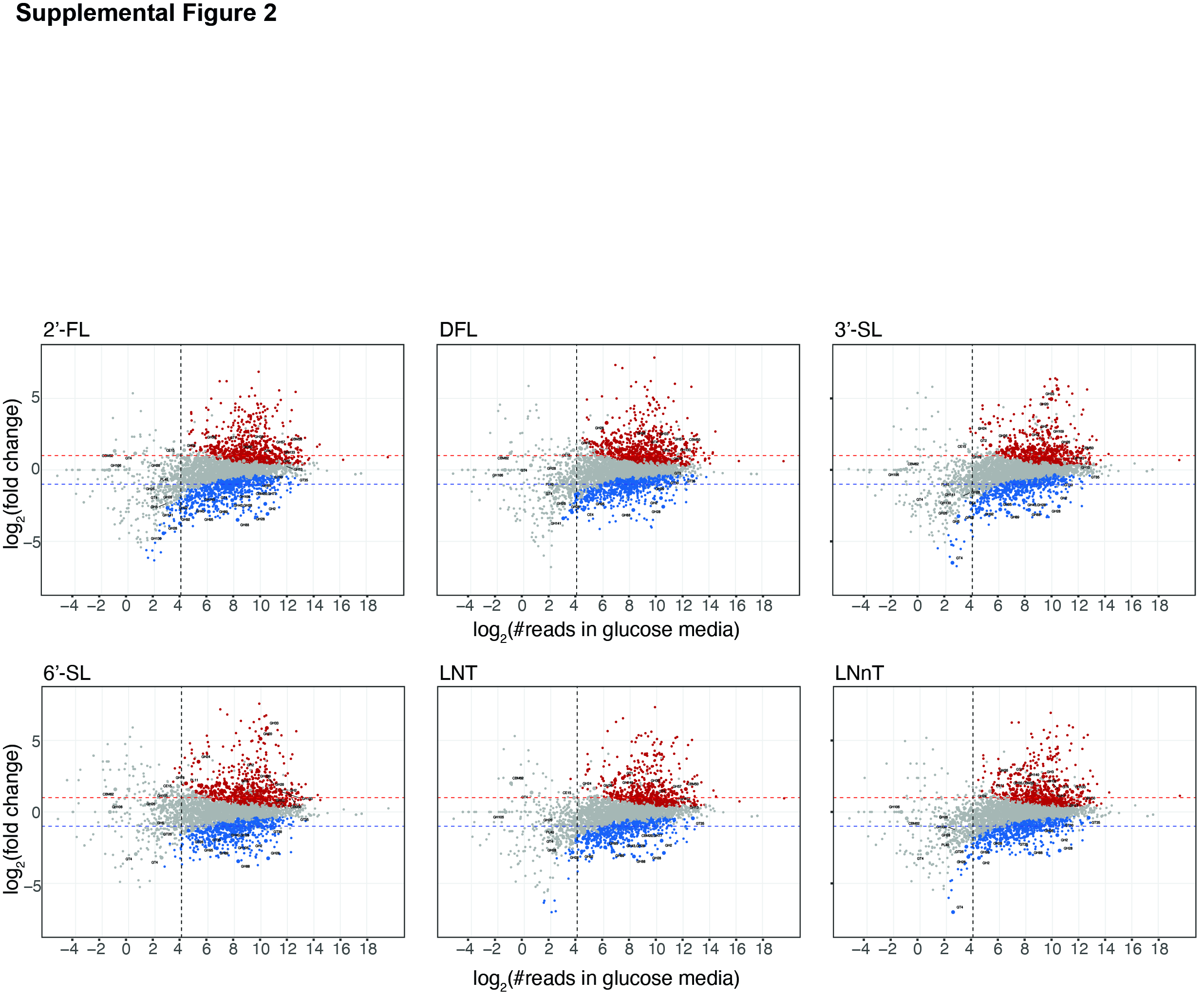

Supplement: Supplementary Figure 2 — MA plots of HMO transcriptional profiles compared to glucose, shown as M (log ratio) on the y-axis and A (mean average) on the x-axis. Here, the log ratio is calculated as the log2 of the change in expression values of the HMOs vs. glucose, and the mean average is of the glucose read counts. Genes from GH families are labeled with larger dots and display the GH family they belong to. The plot highlights up-regulated (red) and down-regulated (blue) genes, using a threshold of 1 for the absolute log2 fold change, and a threshold of 4 for the log2 glucose mean read count. [file Image_2.jpeg]

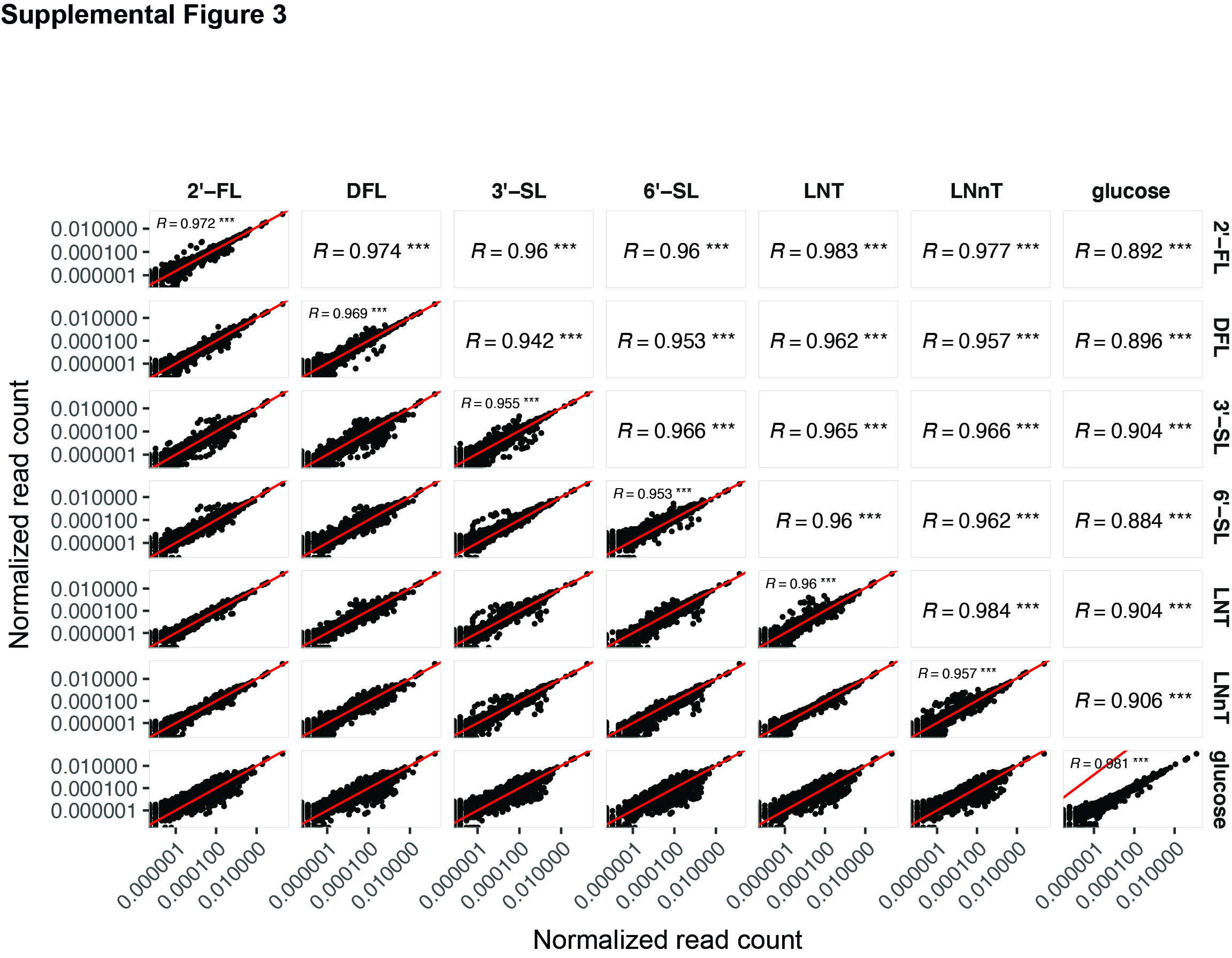

Supplement: Supplementary Figure 3 — B. dorei’s transcriptional profile, as characterized by RNA-Seq experiments, is similar across conditions. The scatterplots display the normalized read count value for all annotated genes in B. dorei’s genome (dots), compared between replicates of the same carbon source (diagonal plots) and between different carbon sources (bottom triangle). Pearson coefficient was calculated for each comparison. [file Image_3.jpeg]
